# Philosophy of Eating

**Published:** 1883-11

**Authors:** 


### PHILOSOPHY OF EATING.

WERE men to exercise the same judgment in reference to their own food that they do in feeding domestic animals, there would be less illness on account of errors of diet. For a matter of such universal importance it has been the subject of many absurd theories.

The world seems to be divided between those who "eat to live" and those who "live to eat." The proper line may be drawn somewhere between these extremes. There is little to choose between a glutton and one who eats too little from a sentimental notion that it is vulgar to eat; and that the less one can eat, and still manage to live, the more refined and spirtual one becomes. If a man has no control over his appetite, and no judgment as to the quantity of food he requires, it would have been better had he belonged to a lower order of animals, subject to the control of a higher intelligence. Neglect or refusal to partake of sufficient food to sustain the body in its full vigor should be regarded as evidence of disease, requiring the attention of a competent physician. Nature will not patiently submit to be abused or cheated.

The quantity and the quality of food required in each individual case depends on the size and health of the person and on his occupation. A person of sedentary habits should regulate the diet to the requirements of the system, remembering that it is safer to err on the side of eating hardly enough than too much. Over-eating produces accumulations of fat, which is a disease of itself, and increases the quantity of blood, rendering one liable to heart disease and apoplexy; and paradoxical as it may seem, insufficient food tends to produce the same diseases. Either condition causes derangements in the circulation that may induce the same troubles.

If we follow the indications of nature we are safe as to foods. What the appetite craves is usually best for us; the stomach notifies us when we require food, and when we have eaten enough. It is often the last mouthful that invites an attack of dyspepsia.

"Variety is the spice of life." In nothing is this more applicable than as to foods. Select a list of foods that experience has taught us are most acceptable, and then from the list get a variety for each day of the week. Salt meats should be used sparingly, because they are more indigestible than fresh. Pies and rich puddings try the digestive organs severely, and cannot be safely indulged in by adults, except they have vigorous outdoor exercise. The quantity and quality of food should depend upon what is required of the individual; just as the amount of fuel requisite depends on the work a steam engine has to perform.

A wise regulation of the food supply can be made to supersede the use of medicines to a very great extent.

The remote cause of a majority of our ordinary ailments is taking cold; the natural functions of the body are retarded, and waste material is retained in the system long enough to do mischief. The usual remedy is to take a cathartic or a laxative in order to remove it. But a more convenient and a more natural plan, in ordinary cases, is to cut off the food supply for twenty-four hours, and trust to nature to do the rest. Instead of food, a few teacupfuls of hot water drank during the day will hasten the desired result. Whenever the bowels become constipated there is an uncomfortable feeling in the system, often accompanied by restlessness and anxiety of mind. The above suggestions, if followed strictly, will bring relief more promptly than medicine, and without its inconveniences.

On the other hand, there is a class of persons who are borne down by constant fear of eating too much, and who are ever anxious lest what they are compelled to eat in order to sustain life may do mischief. It would not be inappropriate to speak of such people as "dietetic cranks." They are probably the most unreasonable and troublesome patients the practicing physician has to deal with. If he favors their theories, there can be no improvement, and if he opposes them he loses their confidence and their patronage. There is as much ill health caused by underfeeding as by overfeeding. The man who has lived too freely may generally moderate his course and regain his health; but through a long course of semi-starvation the digestive organs become weakened, and the stomach contracts so that it cannot properly perform its work; the body cannot be sufficiently nourished. These abnormal conditions generally result from mismanagement during childhood. The child who is restricted to three meals a day comes to the table with a ravenous appetite, and with the certainty that a long fast awaits him after he has finished his repast. These two incentives naturally lead to gluttony.

The child who is permitted to eat whatever it wishes, at all times, becomes the victim of disease, because an unnatural appetite is formed, and it craves those things that derange the digestive organs, but contain but little nourishment. No child was ever injured by plain and wholesome food, no matter how freely or how often it may have been partaken of. When a child is a year old it should be allowed a pretty wide discretion as to foods of this sort. Three meals a day are not sufficient for children. All the operations of the system are more active than with the adult, and the food supply requires more frequent replenishing. Allow children to eat of plain, wholesome food, as often as they desire to, if you wish to escape responsibility for impaired health, which is certain to follow a strict adherence to absurd rules respecting diet.

